# Exploring the Role of Extra Virgin Olive Oil (EVOO) in MASLD: Evidence from Human Consumption

**DOI:** 10.3390/nu17182932

**Published:** 2025-09-11

**Authors:** Melvin Bernardino, Claudio Tiribelli, Natalia Rosso

**Affiliations:** 1 MASLD Unit, Fondazione Italiana Fegato, 34149 Trieste, Italy; ctliver@fegato.it; 2 Department of Life Sciences, University of Trieste, 34100 Trieste, Italy; 3 Philippine Council for Health Research and Development, Department of Science and Technology, Taguig City 1631, Philippines

**Keywords:** metabolic dysfunction-associated steatosis liver disease (MASLD), formerly known as non-alcoholic fatty liver disease (NAFLD), mediterranean diet (MD), extra virgin olive oil (EVOO), hepatic steatosis, anti-inflammation, antioxidant effect

## Abstract

**Background/Objectives:** Metabolic dysfunction-associated steatosis liver disease (MASLD), formerly known as non-alcoholic fatty liver disease (NAFLD) is the leading cause of liver related morbidity and mortality affecting 38% of the adult global population. As of now, there is no clear consensus on a standardized pharmacological treatment for MASLD; therefore, lifestyle interventions particularly diet and exercise remain the first-line approach for both prevention and management. Extra virgin olive oil (EVOO), the primary source of fat in the Mediterranean diet. (MD) is widely recognized as a key contributor to its well-documented health benefits. As a central component of this dietary pattern, EVOO has demonstrated promising therapeutic potential due to its high phenolic content. The primary aim of this review is to synthesize existing human studies examining the effects of olive oil primarily EVOO on key pathological features of MASLD. **Methods:** A systematic search of human clinical and observational studies was conducted across major databases. Key outcomes assessed include hepatic steatosis, inflammation, oxidative stress, fibrosis, liver enzymes, and anthropometric measures. Study quality was evaluated using the Academy of Nutrition and Dietetics Quality Criteria Checklist. **Results:** This review included 25 high-quality studies, 12 of which assessed olive oil alone and 13 evaluated the MD emphasizing extra virgin olive oil (EVOO). EVOO-rich interventions consistently improved hepatic steatosis, liver enzyme levels (ALT, AST), and inflammatory markers in MASLD patients, particularly when paired with calorie-restricted or MD patterns. Benefits were dose- and type-dependent, with EVOO showing superior effects compared to refined olive oils. Modest improvements in lipid profiles and insulin resistance were observed. Longer study durations and higher EVOO intake (>30–50 g/day) yielded greater improvements. Findings suggest EVOO may exert beneficial effects on liver health through its anti-inflammatory and antioxidant properties. Future studies on EVOO’s role in MASLD should use well-characterized oils with known polyphenol and bioactive compound levels and include clear biomarkers of oxidative stress, inflammation, and liver health outcomes on humans. Overall, EVOO represents a promising, non-pharmacological strategy for MASLD prevention and management. **Conclusions:** Current evidence suggests that EVOO, particularly when rich in phenolic compounds, is a promising dietary strategy for managing MASLD due to its hepato-protective effects, especially within a Mediterranean diet framework. However, findings are limited by study heterogeneity and a lack of high-quality randomized controlled trials, highlighting the need for future research to refine optimal dosing, assess long-term outcomes, and clarify underlying mechanisms.

## 1. Introduction

Metabolic dysfunction-associated steatosis liver disease (MASLD), formerly known as non-alcoholic fatty liver disease (NAFLD) is the leading cause of liver-related morbidity and mortality [1]. Currently, MASLD affects approximately 38% of the adult population and 7% to 14% of the pediatric population. Projections indicate that by 2040, the prevalence of MASLD in adults will continue to increase exceeding to 55% [2]. MASLD characterized by the accumulation of excess triglycerides in the liver alongside at least one cardiometabolic risk factors, such as obesity, type 2 diabetes, dyslipidemia, or hypertension, and in the absence of significant alcohol intake or other secondary causes of liver fat accumulation [3]. MASLD encompasses a spectrum of liver conditions, including isolated hepatic steatosis termed metabolic dysfunction-associated Steatotic liver (MASL) as well as metabolic dysfunction-associated steatohepatitis (MASH), and more advanced stages such as fibrosis and cirrhosis [4]. The pathophysiology of MASLD is best explained by the multiple-hit hypothesis, which proposes that liver damage results from the combined effects of metabolic dysfunction, poor diet, sedentary lifestyle, and genetic predisposition acting simultaneously [5].

To date, there is no established consensus on pharmacological therapy for the management of MASLD. As a result, lifestyle modification, particularly dietary interventions and regular physical activity, continues to represent the cornerstone of both prevention and therapeutic management [4,6]. Emerging evidence underscores the critical role of nutritional quality and dietary patterns in modulating metabolic pathways and liver health, positioning diet as a primary modifiable factor in the clinical approach to MASLD [7].

The Mediterranean diet (MD) is a dietary pattern traditionally followed by populations in countries bordering the Mediterranean Sea, and it has garnered substantial scientific attention for its protective effects against a range of chronic non-communicable diseases [7]. This dietary pattern is characterized by a high intake of fruits, vegetables, whole grains, legumes, nuts, and olive oil, moderate consumption of fish and poultry, and low intake of red meat and processed foods. Its benefits are largely attributed to the synergistic effects of nutrient-dense foods rich in dietary fiber, antioxidants, and unsaturated fatty acids, which collectively contribute to a range of physiological mechanisms known to support metabolic and systemic health [8].

Among the core components of the MD, extra virgin olive oil (EVOO) stands out as the principal source of dietary fat and a key contributor to its health-promoting properties. EVOO is rich in monounsaturated fatty acids primarily oleic acid, and contains a wide array of bioactive compounds, including polyphenols, tocopherols, and phytosterols, which are known for their anti-inflammatory and antioxidant effects [9,10,11]. Emerging evidence suggests that EVOO may play a particularly important role in modulating metabolic dysfunction and liver-related disorders, including MASLD [12,13]. Given the current lack of pharmacological consensus for MASLD treatment, dietary interventions such as the incorporation of EVOO within a MD framework present a promising, non-invasive strategy for prevention and management.

This review summarizes the current body of evidence on the beneficial effects of EVOO specifically in the context of MASLD. Given the limited number of clinical studies specifically addressing EVOO’s role in MASLD, this review also considers the potential impact of different types of olive oil, considering variations in different factors, which may influence their biological activity and therapeutic relevance.

The primary aim of this review is to synthesize existing human studies examining the effects of olive oil primarily EVOO on key pathological features MASLD, including hepatic steatosis, oxidative stress, inflammation, and fibrosis. In addition, the review assesses EVOO’s influence on body mass index (BMI), other anthropometric parameters, and biochemical markers related to liver function. Beyond clinical outcomes, this review also compiles detailed information on the dietary context in which EVOO administered, including dosage, duration of intervention, and the specific olive oil cultivars/country employed. This comprehensive approach seeks to identify and clarify the critical variables that may influence the therapeutic efficacy of olive oil primarily “EVOO” on the management of MASLD.

## 2. Materials and Methods

This review aimed at summarizing and critically discussing the current scientific evidence regarding the effects of Olive Oil/EVOO consumption in the context of MASLD. The review primarily focuses on human studies, including clinical trials and observational studies.

### Literature Search Strategy and Data Synthesis

Relevant studies were identified through a comprehensive search of electronic databases including PubMed, Scopus, Web of Science, and Google Scholar. Keywords and search phrases included the following: “Extra Virgin Olive Oil” “Olive Oil” “EVOO” “MASLD” “Metabolic Dysfunction-Associated Steatotic Liver Disease” “MASLD” “Non-Alcoholic Fatty Liver Disease” “NAFLD” “liver health” “fatty liver” “dietary intervention” “Mediterranean Diet” “human study” “clinical trial” “observational”. No date restrictions were applied in the selection of studies.

Studies were included if they met the following criteria: (1) original human research, including randomized controlled trials, quasi-experimental studies, cross-sectional analytical studies, or observational designs, published in English; (2) participants aged 18 years or older with a confirmed diagnosis of MASLD; (3) interventions incorporating olive oil, either within any dietary context or Mediterranean dietary pattern emphasizing EVOO; and (4) reporting relevant liver-related outcomes, including hepatic steatosis, liver inflammation, oxidative stress, liver enzymes, lipid and glycemic profiles, anthropometric measures, or other clinically relevant endpoints. Studies were excluded if they: (1) involved animal or in vitro models, (2) included pediatric populations, or (3) were conference abstracts, reviews, editorials, or other publications without primary data and written in other language other than English.

The quality of the retrieved articles was evaluated using the Academy of Nutrition and Dietetics Quality Criteria Checklist (ANDQCC) for primary research [14]. This is a tool widely used in nutrition and dietetics research to assess study rigor [15,16,17]. This checklist comprises ten questions assessing the validity. It examines both internal and external biases to appraise the rigor of the studies’ inclusion and exclusion criteria, methods of data collection and analysis, and the applicability of the findings, ultimately grading the overall study quality.

The authors manually extracted data from the selected articles, focusing on key outcomes such as hepatic steatosis, inflammation, oxidative stress, liver enzymes, and other liver-related parameters, blood lipid, blood sugar profiles, anthropometrics, and other relevant clinical measures. The findings were synthesized qualitatively, highlighting areas of emerging evidence as well as gaps in the existing literature. Discrepancies arising during study selection, data extraction, or quality appraisal were addressed through deliberation among the review team and by referring to the underlying evidence.

In this review, the term MASLD is used by the updated nomenclature [18,19,20]. However, it is acknowledged that the findings also apply to NAFLD, as this was the term predominantly used in the existing literature.

## 3. Results

The review encompassed 12 research articles that directly evaluated the effects of various types of olive oil on MASLD alongside 13 studies examining the impact of the MD, with a particular emphasis on EVOO as the primary dietary fat source. In total, 25 studies received a positive quality rating based on the validity criteria outlined in the ANDQCC, reflecting a consistently high methodological standard across the evidence base as shown in Table 1.

The evaluation of study quality indicates a positive and reliable body of evidence. Included studies demonstrated strong methodological design, with clear research questions, unbiased participant selection, well-matched comparison groups, and rigorous statistical analyses. These strengths contribute to the confidence in the observed improvements in hepatic and metabolic outcomes. While some studies did not report details on withdrawal handling or employ blinding, these limitations are understandable given the challenges inherent in dietary intervention research. Importantly, the consistent positive ratings across the majority of studies suggest that these factors did not substantially compromise the validity of findings. The comprehensive and transparent reporting in many studies further enhances the credibility of the evidence.

### 3.1. Olive and MASLD

Olea europaea L., commonly referred to as the olive tree, is a small tree species predominantly found in Mediterranean regions [13]. Its primary derivative, “olive oil”, has gained considerable attention in recent years, not only for its distinctive sensory qualities but also for its well-documented health-promoting properties. EVOO is widely used in the human diet, especially in the MD and has long been renowned for its many health-promoting properties. This review compiles human studies that have directly evaluated various types of olive oil in the context of MASLD, as summarized in Table 2.

#### 3.1.1. Effects of Olive Oil on Hepatic Steatosis, Inflammation, and Oxidative Stress

Multiple randomized clinical trials and intervention studies consistently demonstrated that olive oil supplementation, particularly EVOO rich in phenolic compounds, significantly reduces hepatic steatosis in patients with MASLD. Patti et al. (2020) [23] and Pintó et al. (2019) [24] reported the use of EVOO within the context of MD. Rezaei et al. (2019) [21], Sofi et al. (2010) [22], and Nigam et al. (2014) [25] investigated the effects of what is referred to as “standard olive oil.” Among these, only Rezaei et al. (2019) [21] provided detailed information on the nutrient composition, noting a relatively high monounsaturated fatty acid (MUFA). MUFA has been suggested to be beneficial for human health, particularly by improving blood lipid profiles by raising High Density Lipoprotein (HDL) also known as “good” cholesterol and lowering Low Density Lipoprotein (LDL) also known as “bad” cholesterol [46]. Furthermore, all studies reported a reduction in hepatic steatosis, as assessed through imaging techniques.

The anti-inflammatory effects of olive oil are notable, especially with “high-oleocanthal EVOO,” which decreases pro-inflammatory cytokines including Interleukin-6 (IL-6), Interleukin-17A (IL-17A), Tumor Necrosis Factor-alpha (TNF-α) and Interleukin-1 beta (IL-1β) while increasing the anti-inflammatory cytokine Interleukin-10 (IL-10) [23].

The antioxidant effect was assessed through oxidative stress markers, with Sofi et al. (2010) [22] found no significant changes in lipid peroxidation (TBARS) or reactive oxygen metabolites and Rezaei et al. (2019) [21] noted no significant difference in total antioxidant capacity and malondialdehyde. Both studies reportedly used “olive oil” but did not provide information on its polyphenol content or other bioactive compounds.

Kruse et al. (2020) [26] reported conflicting results following an eight-week daily supplementation of “refined olive oil” in obese men with MASLD who followed an isocaloric diet. The study, which involved an approximate intake of 50 g per day, found an increase in hepatic steatosis and no significant changes in inflammation markers such as IL-6, IL-8 and Cytokeratin-18 (CK-18). Furthermore the study by Shidfar et al. (2018) [28] observed no improvement in hepatic steatosis among overweight MASLD patients. However, it did report improvements in Aspartate Aminotransferase (AST) and Alanine Aminotransferase (ALT) liver enzyme. Unlike Kruse et al. (2020) [26] this study did not prescribe a specific dosage of “virgin olive oil”; instead, it required that “virgin olive oil” constitute at least 20% of the participants’ total energy intake per day, making “virgin olive oil” the primary fat source in a hypocaloric diet.

These findings suggest that the effects of olive oil on hepatic steatosis, as well as its anti-inflammatory and antioxidant benefits, may depend on several factors particularly the dosage, the type of olive oil used, and the duration of the intervention. EVOO, characterized by its high phenolic content, appeared to be especially beneficial. Overall, olive oil demonstrates clear potential in reducing hepatic steatosis and inflammation, both of which are key contributors to the progression of MASLD. However, due to the limited number of well-designed, large-scale clinical trials, further research is warranted. Additional studies are needed to explore the anti-inflammatory and antioxidant effects of EVOO in human populations.

#### 3.1.2. Effects of Olive Oil on Liver Enzymes

Liver enzyme profiles such as ALT and AST are important markers of hepatic injury and inflammation. Most studies reviewed demonstrated significant reductions in ALT and AST following olive oil supplementation. For instance, Rezaei et al. (2019) [21], Sofi et al. (2010) [22], and Patti et al. (2020) [23] all reported decreases in these enzymes after 2 to 12 months of intervention, indicating improved liver function. Gamma-glutamyl transferase (GGT), another enzyme linked to liver health, showed less consistent improvements, with only a few studies reporting reductions [22,27]. Interestingly, the WELCOME study in the United Kingdom [32] observed reductions in ALT and AST without corresponding improvements in hepatic steatosis, The overall trend supports that olive oil can reduce liver enzyme elevations commonly seen in MASLD.

#### 3.1.3. Effects on Blood Lipid and Glucose Profiles

Olive oil’s influence on blood lipids and glycemic control presents a mixed but promising picture in the context of MASLD. Several studies noted significant decreases in serum triglycerides (TG), particularly when olive oil was combined with Hypocaloric or Mediterranean diets [21,25,27,31]. Improvements in LDL cholesterol and HDL cholesterol levels were less consistent; Sofi et al. (2010) [22] and Nigam et al. (2014) [25] documented beneficial LDL reductions and HDL increases, but many studies reported no significant changes in these parameters. Glycemic markers such as fasting blood glucose and glycated hemoglobin (HbA1c) largely remained stable, except for notable improvements in insulin resistance indexes (HOMA-IR) [25,30]. Taken together, olive oil exerts modest but favorable effects on lipid metabolism and insulin resistance, particularly in combination of lifestyle modification.

#### 3.1.4. Effect of Olive on Anthropometric and Clinical Outcomes

Reductions in body weight, BMI, and waist circumference (WC) are commonly observed in MASLD patients consuming olive oil alongside a hypocaloric diet or MD. Studies by Rezaei et al. (2019) [21], Patti et al. (2020) [23], and Keshk et al. (2022) [31] demonstrated consistent decreases in these anthropometric markers after interventions ranging from 2 to 6 months, reflecting the role of olive oil role in weight management and central adiposity reduction. Conversely, in the study of Scorletti et al. (2014) [32] and Kruse et al. (2020) [26] among obese MASLD patients who followed isocaloric diets reported minimal or no changes in weight, BMI and WC.

Interestingly, only one study reported improvements in blood pressure, suggesting limited cardiovascular parameter modulation independent of weight loss [21]. Overall, olive oil contributes positively to body composition in the context of MASLD primarily when integrated into a calorie-controlled dietary pattern.

#### 3.1.5. Dose and Type of Olive Oil

As of today, the European Food Safety Authority (EFSA) has declared that the protective effects of EVOO can be achieved through a daily intake of at least 20 g of EVOO or 5.0 mg of hydroxytyrosol (HT) and its derivatives (such as oleuropein complex and tyrosol) [47].

The dose and type of olive oil used across studies significantly influence the outcomes observed in MASLD patients. The available study employed daily doses ranging from approximately 6.5 mL to 60 mL.

Pintó et al. (2019) [24] administered approximately 60 mL/day of “EVOO”, which was rich in phenolic compounds. After a three-year intervention, conducted following a Mediterranean diet, the study observed a significant reduction in hepatic fat prevalence. The participants were older adults at high risk of cardiovascular disease (CVD), most of whom were overweight. In contrast, as mentioned previously, Kruse et al. (2020) [26] used a dosage 50 mL/day of “refined olive oil” in obese men, but found no significant changes in hepatic steatosis, inflammatory markers, blood lipid profiles, blood glucose levels, or anthropometric measurements.

Moreover, Patti et al. (2020) [23] administered 32 mL of high-oleocanthal EVOO for 2 months, reporting marked anti-inflammatory effects alongside steatosis reduction. The type of olive oil such as refined, virgin, or extra virgin olive oil have is an important factor. Based on the available studies “EVOO”, characterized by higher phenolic content, consistently showed superior effects on liver health markers and inflammatory cytokines compared to “refined oils”. Additionally, studies used olive oil enriched with polyunsaturated fatty acids (PUFAs), such as Eicosapentaenoic Acid (EPA) and Docosahexaenoic Acid (DHA) [22], which may confer added anti-inflammatory benefits.

Overall, both the quantity and quality of olive oil are critical factors that influence its effectiveness in the dietary management of MASLD, with EVOO standing out due to its high phenolic content. The quality of olive oil may play an important role in its effects on MASLD. EVOO, which retains higher levels of phenolic compounds, appears to provide more favorable outcomes in several studies, particularly in relation to steatosis, liver enzymes, and inflammatory markers. In contrast, refined olive oils, which have lower levels of these bioactive components, seem to offer benefits mainly as a source of MUFA, and in some cases show limited or no improvement in liver fat unless paired with dietary restriction. While more evidence is needed to confirm these distinctions, current findings suggest that EVOO could be preferable to refined oils for supporting liver health.

These findings underscore the importance of not only selecting high-quality olive oil, such as EVOO, but also determining the optimal dosage needed to achieve beneficial effects on liver health. However, given the limited number of well-designed studies specifically focusing on “EVOO”, further research is needed to validate its therapeutic potential, elucidate underlying mechanisms, and establish evidence-based recommendations for its clinical use in MASLD management. Moreover, there is a need to further investigate the specific quantity and composition of phenolic compounds responsible for these effects, as well as to establish their safe and effective intake levels about liver health particularly on MASLD.

#### 3.1.6. Dietary Context and Study Duration

Dietary context and intervention duration play pivotal roles in determining the impact of olive oil supplementation on MASLD outcomes. Most studies incorporating olive oil into hypocaloric diet or MD reported greater improvements in liver fat, liver enzymes, and anthropometric measures compared to those with isocaloric or habitual diets. Rezaei et al. (2019) [21] and Keshk et al. (2022) [31] paired olive oil supplementation with calorie restriction following MD pattern, leading to significant weight loss and hepatic steatosis reduction. Conversely, Kruse et al. (2020) [26] administered olive oil with an isocaloric diet and found no improvement in liver fat; in fact, intrahepatic lipid increased, highlighting the importance of caloric deficit or dietary quality in driving benefits.

Duration also varied widely from 8 weeks (Kruse et al., 2020) [26] to 6 years (Cueto-Galán et al., 2017) [29] with longer interventions generally providing more improvements. Shorter studies, such as Sofi et al. (2010) [22] and Patti et al. (2020) [23], still reported favorable outcomes but sometimes limited to biochemical markers rather than full clinical improvements. Study duration impacts outcomes. Short-term studies highlight olive oil’s rapid improvements in liver enzymes and steatosis that often occur alongside weight loss. Medium-term studies confirm metabolic improvements. Long-term trials show sustained liver protection, even without ongoing changes in weight or blood chemistry.

The olive oil’s beneficial effects on the context of MASLD optimized within supportive dietary pattern and require adequate intervention periods for maximal impact. In this light, this review also collected studies that assess the effect of MD on MASLD highlighting EVOO as the primary source of fat in the diet.

### 3.2. Olive Oil as a Core Component of the Mediterranean Diet and Its Clinical Relevance for MASLD Dietary Management

Emerging evidence underscores MD, particularly when enriched EVOO, as a cornerstone in managing MASLD. The collective findings from a spectrum of randomized controlled trials, prospective cohorts, and observational studies consistently show the beneficial impact of EVOO-rich MD on hepatic fat accumulation, inflammation, oxidative stress, metabolic parameters, anthropometric and other related clinical outcomes. as summarized in Table 3 and Table 4.

#### 3.2.1. Hepatic Steatosis and Liver Function Markers

Across nearly all studies reviewed, higher adherence to the MD characterized by daily EVOO consumption was associated with significant reductions in intrahepatic fat content (IFC) and hepatic steatosis indices. Quetglas-Llabrés et al. (2024) [33] and Montemayor et al. (2022) [39] observed significant reductions in IFC and visceral fat with higher MD adherence. Properzi et al. (2018) [40], Marin-Alejandre et al. (2019) [36], Kaliora et al. (2019) [37] and Abbate et al. (2021) [43] similarly reported decreased hepatic steatosis content following EVOO-rich MD interventions in a randomized clinical trial.

Liver enzymes such as AST, ALT, and GGT considered surrogate markers of liver inflammation and injury, consistently improved across studies emphasizing EVOO. High EVOO intake within MD regimens contributed to reductions in AST, ALT, and GGT in both controlled trials conducted by Marin-Alejandre et al. (2019) [36], Ristic-Medic et al. (2021) [35] Quetglas-Llabrés (2024) [33] and observational data from Barrea et al. (2023) [38].

#### 3.2.2. Inflammation, Oxidative Stress, and Fibrosis

The olive oil’s antioxidant capacity largely attributed to its polyphenol content appears to be a central mechanism in modulating oxidative stress and inflammatory responses. Studies such as Quetglas-Llabrés et al. (2024) [33] demonstrated marked increases in antioxidant enzymes such as Catalase (CAT), Superoxide Dismutase (SOD), Glutathione Peroxidase (GPx), Glutathione Reductase (GRd) and reduced lipid peroxidation such as lower Malondialdehyde (MDA) and Oxidized Low-Density Lipoprotein (oxLDL) in high MD adherence groups. The upregulation of Toll-Like Receptor 4 (TLR4) expression, observed in the same study, may reflect immunomodulatory activity mediated by this EVOO-enriched dietary pattern.

Likewise, studies reported reductions in C-reactive protein (hs-CRP) [35,36,37] and other inflammatory cytokines such as visfatin and leptin [37], indicating attenuation of chronic low-grade inflammation. A few studies also noted improvement in liver fibrosis scores [37], although changes in liver stiffness were mostly nonsignificant [37,40,43,48]. Interestingly, in a study involving patients with biopsy-confirmed MASLD [44], individuals without significant liver inflammation and fibrosis demonstrated greater adherence to the MD. Increased adherence to this dietary pattern was associated with a reduced likelihood of developing high-grade hepatic steatosis. Moreover, patients exhibiting lower grades of steatosis presented with elevated circulating levels of adiponectin, a hormone known for its anti-inflammatory and insulin-sensitizing properties [49].

#### 3.2.3. Glycemic Control, Insulin Resistance and Lipid Profile

Consistent with its metabolic benefits, EVOO consumption within the MD framework showed a significant improvement in glycemic control and insulin sensitivity. Studies reported lower fasting glucose, insulin levels, and HOMA-IR, suggesting that EVOO-enriched MD contributes to enhanced glucose metabolism an essential component in MASLD which is closely linked to insulin resistance [35,36,42].

Most of the studies consistently observed reductions in TG, TC, and LDL, with concurrent increases in HDL following or high adherence to that EVOO-enriched MD [33,35,36,42].

**Table 3:** Summary of Interventional Studies Assessing the effect of Mediterranean Diet on Metabolic Associated Steatotic Liver Disease (MASLD).

Study (Author Year and Country)	Type and Duration of Study	Participants Characteristics	Intervention/Grouping and Comparator/Control	Incorporation of Olive Oil	Effects of Mediterranean Diet			
					Hepatic Steatosis, Inflammation and Oxidative Stress	Liver Parameters	Blood Lipid and Blood Sugar Profile	Anthropometric and Clinical Measurements
**Quetglas-Llabrés et al., 2024;** **Spain** [33]	Prospective Randomized Trial; 2 years intervention	40 adult patients diagnose with MASLD and Metabolic syndrome; 40–60 years old; average BMI of 32 kg/m^2^ Magnetic Resonance Imaging; Intrahepatic Fat Content	Low Adherence to Mediterranean Diet vs. High Adherence to Mediterranean Diet	Adherence to Mediterranean Diet 17 item questionnaire; [50] Use only EVOO for cooking, salad dressings, and spreads.	↓ Intra Hepatic Fat Content (IFC) on High Adherence Group On High Adherence Group ↑ CAT ↑ SOD ↑ GPx ↑ GRd ↑ GSH level in erythrocytes ↓ MDA ↓ oxLDL ↑ TLR4 expression	High Adherence Group: ↓ AST ↓ ALT ↓ GGT ↓ Cytokeratin-18 plasma levels	Both group ↓ LDL ↓ TC ↓ TG ↑ HDL High Adherence Group: ↓ Glycemia ↓ HbA1C	Both group ↓ BMI High Adherence Group: ↓ Body Fat % ↓ Visceral Fat
**George et al., 2019;** **Australia** [48]	Multicenter Randomized Controlled Trial: 12 week intervention	18 adult patients with MASLD; male/female; average age of 52 years old; average BMI of 31.6 kg/m^2^ Ultrasound or liver biopsy; Intrahepatic Lipids Liver stiffness measurement (Fibroscan)	Mediterranean Diet vs. Low fat Diet	Adherence to Mediterranean Diet 14 item questionnaire [51] Extra virgin olive oil > 4 tablespoon Mediterranean Diet Group were given supply of EVOO and nuts	↔ Intrahepatic Lipids Non-significant but Mediterranean Diet group showed 8% reduction ↔ Liver Stiffness Measurement ↔ hs-CRP levels	↔ AST ↔ ALT ↔ GGT	↔ TG ↔ TC ↔ LDL ↔ HDL ↔ HOMA-IR	↔ BMI ↔ Weight ↔ WC ↓ Visceral Fat
**Ristic-Medic et al., 2021;** **Serbia** [35]	Randomized Controlled Trial: 3 months	12 adult MASLD patients; all male; 27–42 years old; BMI of 25 to 35 kg/m^2^; Liver ultrasound and Fatty Liver Index	Calorie Restricted Mediterranean Diet vs. Low fat Diet	EVOO as the principal fat, ensuring total fat intake comprised up to −35% of total energy	↓ Hepatic Steatosis Index ↓Fatty Liver Index ↓ lipid accumulation index ↓ hs-CRP levels	↓ AST ↓ ALT ↓ GGT	↓ TG ↓ TC ↓ LDL ↓ TG-HDL ratio ↑ HDL ↓ Fasting Glucose ↓ Insulin ↓ HOMA IR	↓ BMI ↓ WC ↓ Body Fat % ↓ Visceral Fat
**Marin-Alejandre et al., 2019;** **Spain** [36]	Randomized Controlled Trial: Fatty Liver in Obesity (FLiO); 6 months intervention	39 adult MASLD patients; male/female; age 40–80 years old BMI 27.5 kg/m^2^ to <40 kg/m^2^; abdominal ultrasonography	Personalized Dietary Strategies; Fatty Liver in Obesity (FLiO) characterized by high adherence to the Mediterranean Diet (MedDiet) vs. Control diet based on American Heart Association (AHA) guidelines	FLiO Diet emphasize the used of extra virgin olive oil as a primary fat source Adherence to Mediterranean Diet 17 item questionnaire; [50] Use only EVOO for cooking, salad dressings, and spreads.	↓ hepatic volume and hepatic fat content ↔ Liver Stiffness Measurement ↓ hs-CRP levels ↑ adiponectin levels ↑ Total Antioxidant Capacity (TAC) of the Diet	↓ AST ↓ ALT ↓ GGT	↓ TG ↓ TC ↓ LDL ↓ TG-HDL ratio ↑ HDL ↓ Fasting Glucose ↓ Insulin ↓ HOMA IR	↓ BMI ↓ WC
**Kaliora et al., 2019;** **Greece** [37]	Prospective Non Randomized Intervention Trial	44 adult patients with MASLD with nonsignificant fibrosis; male/female; 18 years of age and above BMI > 25 kg/m^2^; Abdominal ultrasound (US) and elastography ultrasound stiffness	Mediterranean diet	Emphasis was given to use EVOO as the main fat in diet. Adherence to Mediterranean diet [52]	↓ liver fibrosis score/hepatic steatosis ↓ C-reactive protein (CRP), ↓ oxLDL ↔ IL-6 ↔ TNF-a ↔ leptin	↔ AST ↔ ALT	↓ fasting glucose, ↓ HbA1c ↓ visfatin,	↓ BMI ↓ WC ↓ Body Fat ↓ Weight ↓ blood pressure,
**Montemayor et al., 2022;** **Spain** [39]	Multi-center prospective randomized trial; 6 months intervention	138 adult patients with MASLD with Metabolic Syndrome; aged 40 to 60 years old; BMI 27–40 kg/m^2^; Magnetic Resonance Imaging Intrahepatic Fat Content	Mediterranean Diet adherence changes after 6-month: No changes in adherence, Moderate Changes in Adherence and High Changes in Adherence	Adherence to Mediterranean Diet 17 item questionnaire: [53] How much olive oil do you consume per day >4 tablespoon	Increase Adherence to Mediterranean Diet showed in ↓ IFC	Increase Adherence to Mediterranean leads to: ↓ AST ↓ ALT ↓ GGT	↓ TG in moderate adherence group Increase Adherence to Mediterranean leads to: ↓ HOMA-IR	Increase Adherence to Mediterranean Diet showed larger reduction in BMI, WC and Blood Pressure
**Properzi et al. 2018;** **Australia** [40]	Prospective Randomized Trial; 12 weeks trial;	24 adult patients with MASLD; male/female; average age of 51 years old; average BMI of 31.5 kg/m^2^; MRS/proton density fat fraction (MRS-PDFF)	Ad libitum isocaloric diets Mediterranean vs Low fat	750 mL of olive oil for the MD group supply every visit	↓ Hepatic Steatosis/Hepatic triglyceride content ↔ Liver Stiffness Measurement	↓ ALT ↓ GGT	↓ TG ↓ TC ↓ HbA1c	↔ BMI ↔ Weight
**Katsagoni et al., 2018;** **Greece** [41]	Randomized Controlled Trial: 6 months	21 patients in the Mediterranean lifestyle group (MLG) with MASLD; and 21 patients in the Mediterranean diet group (MDG) adult with MASLD males/females; median ages 44 and 48 years old; median BMI 31.67 and 32.44 Liver ultrasound	Control group (CG), (B) Mediterranean diet group (MDG) or (C) Mediterranean lifestyle group (MLG).	Emphasis was given to use EVOO as the main fat in diet. Adherence to Mediterranean diet [52]	All groups: ↔ MASLD fibrosis score There is an improvement in liver function tests and liver stiffness measurement in the MLG	↓ ALT in MLG ↓ GGT in MLG	↔ TG ↔ TC ↔ LDL ↔ HDL Fasting glucose and insulin resistance (HOMA-IR) significantly improved in the Mediterranean lifestyle group.	↓ BMI ↓ Weight for MDG and MLG
**Abbate et al., 2021** **Spain** [43]	Randomized Controlled Trial: 6 months	43 adult patients with MASLD and Mets (MD-HMF) 43 adult patients with MASLD and Mets (MD-PA) male/female; aged 40 to 60 years; BMI between 27 and 40 kg/m^2^; Liver ultrasound	Conventional Diet (CD) group, which followed the American Association for the Study of Liver Disease (AASLD) recommendations Mediterranean Diet–high meal frequency (MD-HMF) Mediterranean Diet–physical activity (MD-PA)	Adherence to Mediterranean Diet 17 item questionnaire: [53] How much olive oil do you consume per day >4 tablespoon Consumption of at least 30 g per day of Olive Oil	↓ intrahepatic fat content/hepatic steatosis across all Mediterranean diet groups ↔ Liver Stiffness Measurement in all group	↓ ALT ↓ GGT	↓ TG ↔ TC ↔ LDL ↑ HDL ↓ HOMA-IR	↓ BMI ↔ WC

↑ increase; ↓ decrease; ↔ did not change; EVOO: Extra Virgin Olive Oil; IFC: Intra Hepatic Fat Content Non-alcoholic fatty liver disease; MD: Mediterranean Diet; BMI: Body Mass Index; WC: Waist Circumference; TG: Triglyceride; TC: Total Cholesterol; LDL: Low Density Lipoprotein; HDL: High Density Lipoprotein; AST: Aspartate aminotransferase; ALT: alanine aminotransferase; GGT: Gamma-Glutamyl Transferase; HOMA-IR: Homeostatic Model Assessment-Insulin Resistance; hs-CRP; high-sensitivity C-reactive protein; MDA: Malondialdehyde; oxLDL: Oxidized Low-Density Lipoprotein; CAT: Catalase; SOD: Superoxide Dismutase; GPx: Glutathione Peroxidase; GRd: Glutathione Reductase; GSH: Reduced Glutathione; TLR4: Toll-Like Receptor 4; HbA1c: Glycated Hemoglobin.

**Table 4:** Summary of Observational Studies Assessing the effect of Mediterranean Diet on Metabolic Associated Steatotic Liver Disease (MASLD).

Study (Author Year and Country)	Type and Duration of Study	Participants Characteristics	Intervention/Grouping and Comparator/Control	Incorporation of Olive Oil	Effects of Mediterranean Diet			
					Hepatic Steatosis, Inflammation and Oxidative Stress	Liver Parameters	Blood Lipid and Blood Sugar Profile	Anthropometric and Clinical Measurements
**Barrea et al., 2023;** **Italy** [38]	Cross Sectional Observational Study	336 adult patients with 46% prevalence of MASLD; male/female; average age of 35 years old; average BMI 31.18 kg/m^2^; Fatty Liver Index	Degree of Adherence: Low, Medium, and High Adherence to Mediterranean Diet	Adherence to Mediterranean Diet 14 item questionnaire [51] EVOO > 4 tablespoons	FLI was significantly higher in subjects with low adherence to MD than subjects with average and high adherence to MD.	Low adherence to MD had significantly ↑ AST, ALT, and GGT than subjects with average and high adherence to MD.	Low adherence to MD had significantly ↑ fasting plasma glucose, fasting plasma insulin, LDL cholesterol, and TG, than subjects with average and high adherence to MD	Visceral adipose index (VAI) was significantly higher in subjects with low adherence to MD than subjects with average and high adherence to MD
**Gelli et al., 2017;** **Italy** [42]	Observational study; 6 months	46 adult patients with MASLD; male/female; 26–71 years old; BMI range of 18.9–45.3 kg/m^2^; Liver Ultrasound	Mediterranean Diet Before vs. after the intervention	Emphasis was given to use EVOO as the main fat in diet. Adherence to Mediterranean diet [52]	↓ liver fat severity/hepatic steatosis	↓ AST ↓ ALT ↓ GGT	↓ TG ↑ HDL ↓ Serum Glucose ↓ HOMA-IR Improvement in total-Chol/HDL, LDL/HDL, TG/HDL ratios and Atherogenic Index of Plasma (AIP)	↓ BMI ↓ WC ↓ Waist to Hip Ratio
**Aller et al., 2015** **Spain** [44]	Observational-Association Study	82 patients with MASLD; male/female; average age 44.2 years old; average BMI of 32.9 kg/m^2^; Percutaneous liver biopsy	Adherence to Mediterranean diet	Adherence to Mediterranean Diet 14 item questionnaire [51] EVOO > 4 tablespoons	Higher adherence to Mediterranean diet leads to higher odds of protect from high grade of steatosis; Patients without liver inflammation and fibrosis tend to exhibit higher adherence to the Mediterranean diet. Higher levels of adiponectin were observed in patients with lower-grade steatosis.	Higher adherence to Mediterranean diet ↓ AST ↓ ALT	Higher adherence to Mediterranean diet ↓ LDL ↑ HDL ↓ degree of insulin resistance	Higher adherence to Mediterranean ↓ BMI ↓ Weight
**Baratta et al., 2017** **Italy** [45]	Observational-Association Study	584 patients presenting with one or more cardiovascular risk factor; male/female; average age 56 years old; average BMI of 30 kg/m^2^; 82.7% of patients of the patients have steatosis; Liver ultrasound	Adherence to Mediterranean diet: Low vs. Intermediate vs. High	Adherence to Mediterranean Diet 14 item questionnaire [51] EVOO > 4 tablespoons	High adherence Mediterranean diet is associated with a lower prevalence of MASLD	Higher adherence to Mediterranean diet ↓ AST ↓ GGT	Higher adherence to Mediterranean diet ↓ TG ↑ HDL ↓ HOMA IR (showing direct association with olive oil intake)	Higher adherence to Mediterranean ↓ BMI

↑ increase; ↓ decrease; EVOO: Extra Virgin Olive Oil; MD: Mediterranean Diet; BMI: Body Mass Index; WC: Waist Circumference; TG: Triglyceride; LDL: Low Density Lipoprotein; HDL: High Density Lipoprotein; AST: Aspartate aminotransferase; ALT: alanine aminotransferase; GGT: Gamma-Glutamyl Transferase; HOMA-IR: Homeostatic Model Assessment-Insulin Resistance; VAI: Visceral adipose index.

#### 3.2.4. Anthropometric and Clinical Outcomes

EVOO-enriched MD interventions led to significant reductions in BMI, WC, body fat percentage, and visceral adiposity across multiple studies in this review. Interestingly in the study of George et al. (2019) [48] there is a decrease in visceral fat without any changes in BMI, WC, hepatic steatosis, inflammation marker, liver markers, blood lipids and blood sugar profile after following an MD for 12 weeks. Furthermore, an observational study claimed that lower adherence to MD showed higher visceral adipose index fatty liver index as compared to subjects with average and high adherence. Likewise in prospective non randomized intervention trial following a MD significantly decreased body fat along with BMI and hepatic steatosis and inflammation markers such as hs-CRP levels and OxLDL [37].

#### 3.2.5. EVOO Dosage and Duration of the Study

The dose and type of olive oil consumption appear to play a pivotal role in mediating the beneficial effects of the MD in individuals with MASLD. Across the reviewed studies, EVOO was the predominant form used, which is notable given its superior nutritional profile particularly its high monounsaturated fat (oleic acid) content and abundant polyphenols with antioxidant and anti-inflammatory properties. The inclusion of EVOO within the MD framework was assessed by evaluating adherence to the MD dietary pattern. This assessment involved specific inquiries regarding the use of EVOO, such as whether it was employed as the primary cooking medium, main culinary fat, or as a dressing for salads and spreads [50,51,52,53].

Studies consistently using EVOO, such as those by Quetglas-Llabrés et al., Marin-Alejandre et al. and Ristic-Medic et al., reported significant reductions in hepatic steatosis, liver enzymes (ALT, AST, GGT), and systemic oxidative stress markers, outcomes not as prominent in studies where the type of olive oil was unspecified. The dosage of EVOO also influenced outcomes, with intakes exceeding 4 tablespoons per day approximately 30–50 g as indicated in the adherence to MD was associated with greater improvements in intrahepatic fat content, HOMA-IR, adiponectin levels, and visceral adiposity. Importantly, EVOO is identified as the primary source of fat within the broader MD, which is rich in antioxidants, plant-based foods, fiber, and healthy fats.

This synergistic dietary matrix likely amplifies the bioactivity of EVOO. Moreover, study duration played a key role: while short-term interventions (≤3 months) demonstrated modest biochemical improvements, more substantial and sustained changes in liver fat content, inflammatory profiles, and metabolic parameters were predominantly observed in studies lasting six months or longer. These findings underscore the importance of sustained intake of EVOO within a comprehensive MD pattern for managing and potentially reversing MASLD.

Several well-designed clinical studies conducted across Spain, Serbia, and Greece have demonstrated that adherence to the MD, particularly when emphasizing EVOO as the primary source of dietary fat, results in significant improvements in individuals diagnosed with MASLD. These studies, which ranged from 12 weeks to 2 years in duration, involved adult participants—typically aged between 40 and 60 years—with overweight or obesity (BMI ranging from 27.5 to 32 kg/m^2^) and confirmed MASLD diagnosed via imaging methods. The MD interventions consistently emphasized the use of EVOO in cooking, salad dressings, and meal preparation.

The incorporation of EVOO, rich in monounsaturated fatty acids (especially oleic acid) and polyphenols in the MD dietary pattern framework, was associated with better liver health outcomes.

#### 3.2.6. Type of Studies

Observational studies consistently demonstrate that higher adherence to the MD, particularly with regular EVOO intake, is associated with lower prevalence and severity of MASLD, improved liver enzymes, better lipid and glucose profiles, and reduced adiposity. Interventional trials, though fewer and often shorter in duration, provide causal confirmation, showing that the MD can reduce intrahepatic fat, improve metabolic and inflammatory markers, and promote weight and visceral fat loss. However, the magnitude of benefit in trials depends on adherence, intervention duration, and whether the diet is combined with broader lifestyle strategies. Taken together, the evidence supports the MD, enriched with EVOO, as both a preventive and therapeutic approach for MASLD. Given the limited number of studies, there is a need to have more well-designed studies.

## 4. Discussion

### 4.1. EVOO as Essential Part of Mediterranean Diet in the Context of MASLD

The term ‘Mediterranean diet’ was first introduced by Ancel Keys in the 1960s, studying the Seven Countries Study. This dietary pattern was associated with significantly lower rates of cardiovascular disease and all-cause mortality over a 15-year follow-up period. As of today, MD is the most extensively studied and globally recognized dietary patterns [54] associated in the prevention and management of CVD [55,56] type 2 diabetes mellitus [57,58], metabolic syndrome [58], kidney disease [59,60,61], neurogenerative diseases [62,63,64], certain types of cancer [65,66] and age related chronic diseases [67].

In the context of adult MASLD, clinical practice guidelines still emphasize following a lifestyle modification such as weight loss, dietary changes, physical exercise and discouraging alcohol consumption [4]. As of now, there is no clear consensus on a standardized pharmacological treatment for MASLD; therefore, lifestyle interventions remain the first-line approach for both prevention and management [68]. While there is broad consensus on the emphasis on consuming plant food products within the MD, it is important to consider that within the framework of a balanced and health-promoting diet, approximately 30% of total energy intake is recommended to be derived from fats, with careful attention to the types and optimal quantities of fats and oils included [69]. Subsequently MASLD involves the pathological accumulation of fat in the liver, there is evident that an isocaloric diet rich in saturated fats may be an important determinant of liver fat accumulation [70]. Therefore, the quality and quantity of dietary fat intake are particularly relevant.

EVOO consists predominantly of triglycerides (97–99%), with a small fraction (1–3%) of minor compounds that are primarily responsible for its biological activity EVOO is particularly rich in MUFA, and contains some PUFA [11]. Studies have shown that EVOO is superior in the management of clinical biomarkers including lowering blood pressure and LDL, increasing protective HDL, improving glycemic control, and weight management in the context of cardiovascular disease. In the context of MASLD this review synthesized data from a wide range of clinical trials and intervention studies evaluating the effect of olive oil, particularly EVOO and related metabolic parameters. Collectively, the evidence underscores the therapeutic potential of EVOO on different outcomes related to MASLD. However, this review highlights that there is variability across studies pointing out the importance of olive oil type, dosage, dietary context, and intervention duration as key modulators of these effects.

Most studies reported significant reductions in hepatic steatosis following olive oil or EVOO supplementation, particularly when integrated into a hypocaloric diet or MD. These reductions are more pronounced in studies employing higher daily doses (≥30 mL/day) of phenolic-rich EVOO over extended durations (≥12 weeks). For example, Pintó et al. (2019) [24] demonstrated a clear decrease in liver fat with long-term (3-year) intake of ~60 mL/day of EVOO. In contrast, Kruse et al. (2020) [26] observed worsening steatosis with refined olive oil at a similar dose, reinforcing the critical role of oil quality specifically, the polyphenol content of EVOO. ALT and AST are key markers of hepatocellular injury were consistently reduced in response to EVOO supplementation across multiple trials. These biochemical improvements occurred both with and without concurrent steatosis reduction, indicating that EVOO may exert direct hepatoprotective effects independent of fat content changes. However, changes in GGT were less consistent, possibly due to its higher variability and sensitivity to alcohol intake, oxidative stress, and cholestasis. EVOO consumption, especially within a MD pattern, exerted favorable effects on serum triglycerides and insulin resistance markers, including HOMA-IR. These effects were potentiated by concurrent caloric restriction, suggesting that the metabolic benefits of olive oil are best realized within structured dietary interventions. However, impacts on LDL, HDL, and fasting glucose were inconsistent, with some trials reporting significant improvements while others showed minimal change. These mixed outcomes may reflect differences in baseline metabolic status, dietary adherence, and intervention duration.

Furthermore, population studies also claimed the protective effect of consumption of EVOO in MASLD risk. In the Multicenter Italian Study on Cholelithiasis (MICOL) study [71], a prospective analysis involving 2436 middle-aged and older adults, evaluate whether dose-increased consumption of EVOO is associated with a lower prevalence of MASLD and if these effects vary based on body weight. They found out that a higher intake of EVOO was found to be associated with a decreased prevalence of MASLD and improved metabolic parameters, especially among individuals classified as overweight or obese. Furthermore, in the same cohort a total of 2754 participants included in the analysis from the MICOL study [72], which aimed to assess the impact of moderate-to-high olive oil consumption on all-cause mortality within a population adhering well to the MD. The findings demonstrate a protective effect of EVOO intake against all-cause mortality. Notably, this protective association persisted despite higher caloric intake. Another noteworthy aspect is the potential gender-specific difference in the protective effects of EVOO consumption. The NUTRIHEP study [73] reported that the protective role of EVOO may vary between males and females. Specifically, the study confirmed that EVOO exerts a protective effect against MASLD predominantly in females, within a Southern Italian cohort characterized by high adherence to the MD. This population-based study provides convincing evidence that EVOO rich in phenolic compounds may reduce hepatic steatosis or lower the risk of developing MASLD, particularly when combined with a hypocaloric or MD in overweight patients. These findings are consistent with the studies included in this review.

Although olive oil is central to the MD, its health effects are not uniform. EVOO is particularly variable in composition depending on the olive cultivar, extraction method, and storage conditions. For example, Picual varieties are typically richer in polyphenols and oleuropein, conferring greater antioxidant capacity than Manzanilla oils, which contain fewer polyphenols but more pigments. Similarly, only mechanically extracted oils preserved through cold pressing retain their full phenolic profile, while refined oils lose much of this bioactive fraction and therefore may be less effective in mitigating hepatic oxidative stress. Storage factors further influence quality; exposure to light, oxygen, and high temperatures accelerates degradation of phenolic compounds, α-tocopherol, and pigments, reducing both shelf life and biological efficacy [11]. The magnitude of EVOO’s protective effect in MASLD depends not only on dietary adherence but also on the quality and stability of the oil consumed. Accordingly, future studies should consider EVOO quality as a critical factor influencing MASLD outcomes.

### 4.2. The Role of Polyphenols in EVOO in the Context of MASLD

There is an increasing evidence that the protective effects of EVOO are likely attributable to its high polyphenol content rather than its monounsaturated fat content. [74] Based on the existing studies EVOO contributes uniquely to the anti-inflammatory, antioxidant, and insulin-sensitizing effects of the MD, likely via synergy with other diet components such as fiber, polyphenols, and omega-3 PUFAs [75]. In the present review there are limited number study showing EVOO exerts anti-inflammatory effects, evidenced by reductions in pro-inflammatory cytokines such as IL-6, TNF-α, IL-1β and increases in IL-10. These outcomes are most robust when EVOO is rich in Oleocanthal. Oleocanthal is a phenolic compound with ibuprofen-like COX-inhibitory activity [76]. Patti et al. (2020) [23] notably demonstrated these effects in just 8 weeks of high-oleocanthal EVOO consumption. Oxidative stress findings were less consistent, possibly due to differences in antioxidant measurement methods and participant baseline status. However, several studies observed the upregulation of endogenous antioxidant enzymes suggesting EVOO may enhance redox homeostasis. Discrepancies in oxidative marker outcomes may also stem from using refined vs. unrefined oils, or insufficient study durations [33].

**Figure 1:**
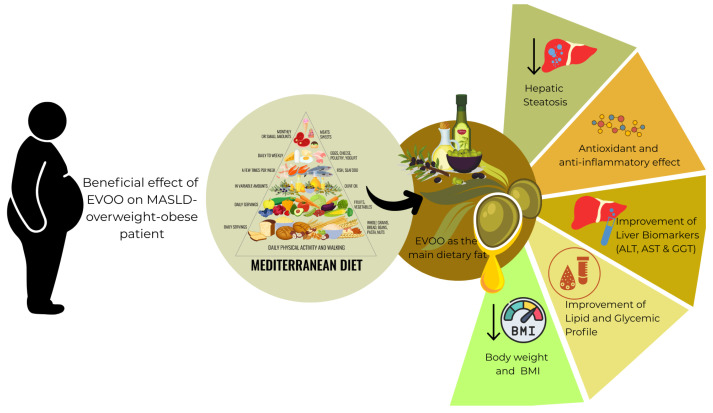
Beneficial effects of extra virgin olive oil (EVOO) on metabolic dysfunction-associated steatotic liver disease (MASLD) in overweight and obese patients within the context of the Mediterranean diet. EVOO as the primary source of dietary fat contributes to multiple health benefits including reduced hepatic steatosis, antioxidant and anti-inflammatory effects, improvement in liver biomarkers (ALT, AST, and GGT), better lipid and glycemic profiles, and decreased body weight and BMI. These findings highlight the therapeutic role of EVOO when incorporated into a Mediterranean dietary pattern for managing MASLD.

Incorporating EVOO regularly within healthy dietary patterns, especially the MD, emerges as a promising nutritional strategy. Figure 1 illustrates the impact of EVOO on MASLD in overweight and obese individuals following a Mediterranean diet. EVOO as the principal fat of the MD, exerts multiple effects relevant to the pathogenesis of MASLD. Its MUFA, particularly oleic acid, improve peripheral insulin sensitivity, regulate hepatic gluconeogenesis and lipogenesis, and reduce Sterol Regulatory Element-Binding Protein (SREBP)-mediated triglyceride accumulation that has been explored on animal models [77]. Additionally it prevents central fat accumulation and modulates adiponectin, enhancing adipose tissue lipolysis and fat redistribution [78]. Beyond lipid metabolism, polyphenols such as oleocanthal, oleuropein, hydroxytyrosol, and tyrosol exert potent antioxidant and anti-inflammatory effects, inhibiting reactive oxygen species, Nuclear Factor kappa-light-chain-enhancer of activated B cells (NF-κB) activation, and leukotriene B4 synthesis. Olive oil additionally improves endothelial function, reduces circulating TNF-α and vascular adhesion molecules, and enhances Glucagon-Like Peptide-1 (GLP-1)–mediated glycemic control, potentially modulating systemic inflammation and the gut-liver axis. Together, these mechanisms link olive oil consumption to improved metabolic and inflammatory profiles, providing a clear rationale for its protective role in MASLD [12].

In this review, this approach is particularly relevant for overweight and obese individuals with MASLD, who are at greater risk of metabolic complications. However, the growing recognition of MASLD in normal BMI individuals often referred to as “lean MASLD” adds complexity to the clinical landscape. Despite its increasing prevalence, lean MASLD remains underexplored, and there is a lack of well-defined clinical guidelines for its management. Emerging evidence suggests that genetic variants and epigenetic modifications may play a significant role in the pathogenesis of MASLD among lean individuals, though the underlying mechanisms have not yet fully explained [79,80]. Understanding how dietary components such as EVOO may interact with these metabolic and genetic profiles could offer novel insights into personalized nutritional strategies for MASLD across different phenotypes. Moreover, the anti-inflammatory and antioxidant properties of polyphenols in EVOO may further contribute to liver health and metabolic improvements, supporting its role as a key component in dietary interventions targeting MASLD even in lean population.

In the context of MASLD, the antioxidant and anti-inflammatory effects of EVOO in human populations remain insufficiently explored and poorly characterized. A key limitation across existing studies is the significant variability in the dosage and composition of the olive oil administered, with many trials failing to quantify or report the phenolic compound content critical bioactive components believed to mediate many of EVOO’s health benefits. As previously mentioned, the EFSA has stated that the protective effects of EVOO can be achieved with a daily intake of at least 20 g of EVOO or 5.0 mg of H) [47]. However, it remains unclear whether these intake levels are equally safe and effective for individuals with MASLD, particularly those who are overweight, obese, or even lean. Future clinical studies should investigate whether these populations require adjusted dosages to optimize therapeutic efficacy while minimizing potential metabolic or hepatic risks.

This lack of standardization not only hampers the ability to draw meaningful conclusions about efficacy but also obscures potential dose–response relationships. Furthermore, the heterogeneity in study design, including differences in duration, participant characteristics, and concurrent dietary interventions, further complicates the interpretation of findings. To fully elucidate the therapeutic potential of EVOO in MASLD, future studies should prioritize the use of chemically characterized EVOO with well-defined concentrations of polyphenols, or preferably, specific bioactive compounds such as simple phenols (e.g., hydroxytyrosol, tyrosol), aldehydic secoiridoids (e.g., oleuropein), flavonoids, and lignans (e.g., acetoxypinoresinol, pinoresinol). Moreover, these studies should incorporate robust biomarkers of oxidative stress and inflammation, alongside liver-specific clinical outcomes.

Nevertheless, accumulating evidence from both clinical trials and observational studies highlights the beneficial effects of EVOO consumption in individuals with MASLD. These studies consistently report reductions in hepatic steatosis, improvements in liver function biomarkers, and favorable changes in blood lipid and glucose profiles, as well as anthropometric measures.

### 4.3. Limitations and Future Direction

Despite encouraging findings, several limitations in the current body of research warrant consideration. A key issue is the variability in olive oil quality, with many studies failings to report the polyphenol content, making it difficult to establish clear dose–response relationships. Additionally, most interventions were short-term, typically lasting three months or less, which limits the ability to assess the long-term hepatic and metabolic effects of EVOO.

There is also substantial heterogeneity in study design, including differences in caloric intake, overall diet composition, and the type and dose of olive oil used, which complicates cross-study comparisons. Moreover, methods used to assess hepatic steatosis vary widely from invasive liver biopsy to non-invasive techniques such as liver ultrasound, magnetic resonance imaging (MRI), controlled attenuation parameter (CAP), and fatty liver index (FLI) adding further variability to outcome evaluation. Another gap in current studies is the limited assessment of inflammatory and oxidative stress markers. Despite growing evidence supporting the role of polyphenols in EVOO as key mediators of its beneficial effects in MASLD. Future studies should consider these limitations.

To address these limitations, future research should prioritize adequately powered, long-term randomized controlled trials using standardized EVOO formulations with clearly defined phenolic profiles and concentrations, while incorporating comprehensive metabolic, hepatic, and inflammatory outcome measures.

## 5. Conclusions

Taken together, the current evidence supports the integration of EVOO particularly when rich in phenolic compounds, as a key dietary strategy for managing MASLD. EVOO demonstrates compelling hepato-protective effects, including reductions in hepatic steatosis, liver enzymes, inflammation, and insulin resistance, particularly when used within a MD framework. However, the available evidence is limited due to study heterogeneity, and the relatively low number of high-quality randomized controlled trials, as well as potential publication bias.

Consequently, future studies should aim to refine optimal dosing, particularly polyphenols, explore long-term outcomes, and elucidate molecular mechanisms to further support clinical recommendations.

## Figures and Tables

**Table 1:** Quality Assessment of Studies Using the Academy of Nutrition and Dietetics Quality Criteria Checklist.

Author and Year of Publication	1	2	3	4	5	6	7	8	9	10	Quality Rating
Rezaei et al. (2019) [21]	**+**	**+**	**+**	**−**	**−**	**+**	**+**	**+**	**+**	**+**	**Positive**
Sofi et al. (2010) [22]	**+**	**−**	**+**	**−**	**−**	**+**	**+**	**+**	**+**	**+**	**Positive**
Patti et al. (2020) [23]	**+**	**−**	**−**	**−**	**−**	**+**	**+**	**+**	**+**	**+**	**Positive**
Pintó et al. (2019) [24]	**+**	**+**	**+**	**+**	**−**	**+**	**+**	**+**	**+**	**+**	**Positive**
Nigam et al. (2014) [25]	**+**	**+**	**+**	**+**	**−**	**+**	**+**	**+**	**+**	**+**	**Positive**
Kruse et al. (2020) [26]	**+**	**+**	**+**	**+**	**−**	**+**	**+**	**+**	**+**	**+**	**Positive**
Tobin et al. (2018) [27]	**+**	**+**	**+**	**+**	**+**	**+**	**+**	**+**	**+**	**+**	**Positive**
Shidfar et al. (2018) [28]	**+**	**+**	**+**	**+**	**−**	**+**	**+**	**+**	**+**	**+**	**Positive**
Cueto-Galán et al. (2017) [29]	**+**	**+**	**+**	**−**	**−**	**+**	**+**	**+**	**+**	**+**	**Positive**
Yahay et al. (2021) [30]	**+**	**+**	**+**	**−**	**−**	**+**	**+**	**+**	**+**	**+**	**Positive**
Keshk et al. (2022) [31]	**+**	**+**	**+**	**−**	**−**	**+**	**+**	**+**	**+**	**+**	**Positive**
Scorletti et al. (2014) [32]	**+**	**+**	**+**	**+**	**+**	**+**	**+**	**+**	**+**	**+**	**Positive**
Quetglas-Llabrés et al. (2024) [33]	**+**	**+**	**+**	**+**	**−**	**+**	**+**	**+**	**+**	**+**	**Positive**
George et al. (2022) [34]	**+**	**+**	**+**	**+**	**−**	**+**	**+**	**+**	**+**	**+**	**Positive**
Ristic-Medic et al. (2021) [35]	**+**	**+**	**+**	**−**	**−**	**+**	**+**	**+**	**+**	**+**	**Positive**
Marin-Alejandre et al. (2019) [36]	**+**	**+**	**+**	**−**	**−**	**+**	**+**	**+**	**+**	**+**	**Positive**
Kaliora et al. (2019) [37]	**+**	**+**	**+**	**−**	**−**	**+**	**+**	**+**	**+**	**+**	**Positive**
Barrea et al. (2023) [38]	**+**	**−**	**§**	**§**	**−**	**+**	**+**	**+**	**+**	**+**	**Positive**
Montemayor et al. (2022) [39]	**+**	**+**	**+**	**+**	**−**	**+**	**+**	**+**	**+**	**+**	**Positive**
Properzi et al. (2018) [40]	**+**	**+**	**+**	**+**	**−**	**+**	**+**	**+**	**+**	**+**	**Positive**
Katsagoni et al. (2018) [41]	**+**	**+**	**+**	**+**	**−**	**+**	**+**	**+**	**+**	**+**	**Positive**
Gelli et al. (2017) [42]	**+**	**−**	**§**	**−**	**−**	**+**	**+**	**+**	**+**	**+**	**Positive**
Abbate et al. (2021) [43]	**+**	**+**	**+**	**+**	**−**	**+**	**+**	**+**	**+**	**+**	**Positive**
Aller (2015) [44]	**+**	**−**	**§**	**§**	**−**	**+**	**+**	**+**	**+**	**+**	**Positive**
Baratta (2017) [45]	**+**	**−**	**§**	**§**	**−**	**+**	**+**	**+**	**+**	**+**	**Positive**

(+) Answer to validity question was YES. (−) Answer to a validity question was NO (§) Not applicable. Based on the validity questions (1) Was the research question clearly stated? (2) Was the selection of study subjects/patients free from bias? (3) Were study groups comparable? (4) Was method of handling withdrawals described? (5) Was blinding used to prevent introduction of bias? (6) Were intervention/therapeutic regimens/exposure factor or procedure and any comparison(s) described in detail? Were intervening factors described? (7) Were outcomes clearly defined and the measurements valid and reliable? (8) Was the statistical analysis appropriate for the study design and type of outcome indicators? (9) Are conclusions supported by results with biases and limitations taken into consideration? (10) Is bias due to study’s funding or sponsorship unlikely? Quality Rating: Positive: Indicates that the report has clearly addressed issues of inclusion/exclusion, bias, generalizability, and data collection and analysis. Negative: Indicates that these issues have not been adequately addressed. Neutral: Indicates that the report is neither exceptionally strong nor exceptionally weak.

**Table 2:** Summary of Interventional Studies Directly Assessing the Effects of Olive Oil on Metabolic Associated Steatotic Liver Disease (MASLD).

Study (Author Year and Country)	Type and Duration of Study	Participants Characteristics for Olive Oil Arm and MASLD Diagnosis	Type and Dose of Olive Oil	Diet During the Study	Effects of Olive Oil			
					Hepatic Steatosis, Inflammation and Oxidative Stress	Liver Parameters	Blood Lipid and Blood Sugar Profile	Anthropometric and Clinical Measurements
**Rezaei et al. (2019)** **Iran** [21]	Randomized Clinical Trial; Olive Oil vs. Sunflower Oil; 12 weeks intervention	26 adult MASLD patients; male/female; ≥18 years old; BMI of: ≥25 kg/m^2^; Liver Ultrasound	20 mL of standard Olive Oil per day; Composition: Oleic acid (64.3%) Linoleic (15.4%) Palmitic (15.2%) Omega 3 fatty acid (1%)	Hypocaloric diet with 500 kcal deficit (10–15% from protein, 30–35% from fat and 50–55% from the carbohydrates)	↓ Hepatic Steatosis (2x reduction vs. sunflower oil) ↔ IL-6 ↔ Serum malondialdehyde ↔ Total antioxidant capacity n	↓ AST ↓ ALT	↓ Serum TG ↔ FBG	↓ Weight ↓ BMI ↓ WC ↓ Blood Pressure
**Sofi et al. (2010)** **Italy** [22]	Randomized Clinical Trial (Pilot study); Olive Oil enriched with PUFA vs. Olive Oil alone; 12 months intervention	6 adult patients with MASLD; male/female; ≥18 years old; average BMI of ≥29.3 kg/m^2^; Liver Ultrasound	6.5 mL of olive oil enriched with *n*-3 PUFA (EPA/DHA)	The usual diet;not standardized or modified by the researchers	↓ Hepatic Steatosis ↑ Adiponectin levels ↔ TBARS ↔ dROMS ↔ BAP ↔ SHp	↓ AST ↓ ALT ↓ GGT	↔ TC ↓ LDL ↑ HDL ↔ Blood Glucose ↔ HOMA	↔ BMI
**Patti et al. (2020)** **Italy** [23]	Intervention study; before vs. after treatment of High Oleocanthal concentration EVOO; 2 months	23 adult patients with Metabolic Syndrome with Hepatic steatosis; male/female ≥18 years old; average BMI of ≥31 kg/m^2^; Ultrasound Examination	32 mL of EVOO Mono cultivar EVOO with High Oleocanthal Concentration	Mediterranean Diet	↓ Hepatic Steatosis ↓ IL6, ↓ IL 17 A, ↓ TNF-a, ↓ IL-1B ↑IL-10	↔ AST ↓ ALT ↔ GGT	↔ TC ↔ TG ↔ LDL ↔ HDL ↔ Glycemia ↔ Hb1AC ↔ HOMA	↓ Weight ↓ BMI ↓ WC
**Pintó et al., 2019** **Spain** [24]	Randomized Control Trial; Bellvitge PREDIMED trial; EVOO + MD vs. Nuts+ MD; Control group: Low Fat Diet; 3 years intervention	34 adult participants high risk of CVD; male/female; older adults with average age of 64 years old; average BMI of ≥28.7 kg/m^2^; NMR imaging	60 mL/day of EVOO (contains high amount of phenolic compound, antioxidants)	Energy Unrestricted Mediterranean Diet	↓ Hepatic Steatosis prevalence ↓ 12-HETE levels ↔ hs-CRP	↔ AST ↔ ALT	↔ TC ↔ TG ↔ LDL ↔ HDL ↔ Fasting glucose	↔ BMI ↔ Weight ↔ WC
**Nigam et al., 2014** **India** [25]	Randomized Control Trial; Olive Oil vs. Canola oil and soybean/safflower; 6 months intervention	30 adult patients with MASLD; all male; average age of 37 years old; average BMI of 27.2 kg/m^2^; Liver Ultrasound	20 mL of Standard Olive Oil per day	Therapeutic Lifestyle Change (TLD diet) with daily energy intake of 15–21% protein, 55–70% carbohydrates, 20% from fats	↓ Hepatic Steatosis ↓ hs-CRP	↔ AST ↔ ALT	↓ TG ↑ HDL ↓ Fasting Insulin ↓ HOMA-IR ↓ HOMA-BCF ↓ Disposition Index	↓ Weight ↓ BMI ↔ WC
**Kruse et al., 2020 (Germany)** [26]	Parallel-group randomized controlled trial; 8 weeks of daily oil supplementation	11 adult with MASLD; all male; aged 18–65 old; BMI of 30–35 kg/m^2^; H-MRS	Refined olive oil 50 g/day (~3.5 tablespoons) an	Isocaloric diet	↑ Intrahepatic lipid (IHL) ↔ IL-6 ↔ IL-8 ↔ CK 18	↔ AST ↔ ALT	↔ TG ↔ TC ↔ LDL ↔ HDL, ↔ apolipoprotein ↔ B serum ↔ glucose, ↔ insulin, ↔ HOMA IR	↔ Weight ↔ BMI ↔ WC ↔ total body fat ↔ Waist-to-hip ratio (WHR) of
**Tobin et al., 2018** **Norway** [27]	Randomized Control Trial; Omega-3 concentrate vs. olive oil (as placebo) 24 weeks intervention	86 adult patients with MASLD; male/female; ≥18 years old; average BMI of 32.4 kg/m^2^; MRI	Olive oil (gel capsules containing 1 g of olive oil)—3 caps daily.	Calorie restricted diet	↓ Hepatic Steatosis	↓ AST ↓ ALT ↓ GGT	↓ TG	↔ BMI ↔ Weight ↔ WC
**Shidfar et al., 2018 Iran** [28]	Randomized Clinical Trial Olive Oil vs. Normal Cooking Oil; 12 weeks intervention	25 adult patients with MASLD; male/female; average age of 45 years old; average BMI of 29.7 kg/m^2^; Liver Ultrasound	20% of the total energy intake virgin olive oil	Hypocaloric diet with an aim of 5% weight reduction within 3 months with 50% carbohydrates, 20% protein and 30% fats	↔ Hepatic Steatosis (but more improvement on olive oil arm vs. the control)	↓ AST ↓ ALT		↓ Weight ↓ BMI ↔WC
**Cueto-Galán et al., 2017** **Spain** [29]	Randomized Clinical Trial; Part of the PREDIMED Malaga Trial; EVOO + MD, EVOO+ Dried Fruits and Nuts, Control Diet: Low Fat Diet 6 years follow up	117 adult participant where 57% of the total sample have MASLD; male/female; average age of 67 years old; average BMI of 29.55 kg/m^2^; Fatty Liver Index	1 L per week (estimation of 143 mL/day consumption)	Mediterranean Diet	↓ Fatty Liver Index vs. the control group indicating reduced steatosis		Fatty Liver Index Calculation includes triglycerides, specific HDL/LDL changes were not separately reported	Minimal change in BMI ↔ WC
**Yahay et al., 2021** **Iran** [30]	Randomized Clinical Trial; Olive Oil vs. Canola and control: sunflower oil, 10 weeks intervention	24 adult female with PCOS; 18–45 years old; average BMI of 28.84 kg/m^2^; Liver Ultrasound	25 mL per day of Olive oil; High MUFA 69.22%), *n*-6 PUFA (~11.20%), *n*-3 PUFA (~0.63%)	Balanced Diet with 45–60% from Carbohydrates, 30–35% from fats and 15–18% from protein	↓ Hepatic Steatosis (Fatty Liver severity measure of the extent of fat deposition in the liver)	↔ SHGB	↔ TC ↔ TG ↔ LDL ↔ HDL ↓ HOMA-IR	↔ BMI ↔ Weight
**Keshk et al., 2022** **Egypt** [31]	Randomized Clinical Trial Hypocaloric Diet with Olive Oil vs. Hypocaloric Diet without Olive Oil; 6 months duration	30 adult patients with MASLD; male/female; ≥18 years old; BMI of 30–40 kg/m^2^ Transient Elastography Fibroscan	20% of the total energy intake from refined olive oil blend	Hypocaloric diet (-500 kcal/day) but following Mediterranean dietary pattern (50% energy from carbs and 20% from protein)	↓ Controlled Attenuation Parameter (but much lower in Hypocaloric Diet without Oilve Oil)	↓ AST ↓ ALT	↓ TC ↓ TG ↔ LDL ↔ HDL	↓ Weight ↓ BMI ↓ WC
**Scorletti et al., 2014** **United Kingdom** [32]	Randomized Clinical Trial, WELCOME study Omacor (DHA/EPA) vs. olive oil (as placebo) 72 weeks intervention	45 adult patients with MASLD; male/female; average age of 54 years old; average BMI of 32 kg/m^2^ magnetic resonance spectroscopy	4 g per day of olive oil; 1 g of olive oil contains 600 mg of oleic acid plus lesser amounts of linoleic, palmitic, stearic, and a-linolenic acids	General dietary advice	↔ Hepatic Steatosis	↓ AST ↓ ALT	↔ TC ↔ TG ↔ LDL ↔ HDL ↔ Hb1AC ↔Fasting glucose ↔ Fasting Insulin	↔ BMI ↔ Weight

↑ increase; ↓ decrease; ↔ did not change; EVOO: Extra Virgin Olive Oil; MASLD: Metabolic associated steatotic liver disease; CVD: Cardiovascular Disease; PCOS: Polycystic Ovary Syndrome; MD: Mediterranean Diet; PREDIMED: Prevención con Dieta Mediterránea; WELCOME study (Wessex Evaluation of Fatty Liver and Cardiovascular markers in NAFLD with Omacor Therapy); NMR: Nuclear Magnetic Resonance Imaging; H-MRS: Hepatic proton magnetic resonance spectroscopy; BMI: Body Mass Index; WC: Waist Circumference; TG: Triglyceride; TC: Total Cholesterol; LDL: Low Density Lipoprotein; HDL: High Density Lipoprotein; AST: Aspartate aminotransferase; ALT: alanine aminotransferase; GGT: Gamma-Glutamyl Transferase; HOMA: Homeostatic Model Assessment; HOMA-IR: Homeostatic Model Assessment-Insulin Resistance; HOMA-BCF: Homeostatic Model Assessment of Beta-Cell Function; TBARS: Thiobarbituric acid-reactive substances; dROMS: Reactive Oxygen Metabolites Test; BAP: Biological Antioxidant Potential; SHp: Protein sulfhydryls; 12-HETE: 12-Hydroxyeicosatetraenoic acid; hs-CRP: high-sensitivity C-reactive protein; SHGB: Sex Hormone-Binding Globulin.

## Abbreviations

The following abbreviations are used in this manuscript:

ALT	Alanine Aminotransferase
ANDQCC	Academy of Nutrition and Dietetics Quality Criteria Checklist
AST	Aspartate Aminotransferase
BMI	Body Mass Index
CAT	Catalase
CVD	Cardiovascular Disease
DHA	Docosahexaenoic Acid
EPA	Eicosapentaenoic Acid
EVOO	Extra Virgin Olive Oil
GGT	Gamma-Glutamyl Transferase
(GLP-1)	Glucagon-Like Peptide-1
GPx	Glutathione Peroxidase
GrR	Glutathione Reductase
HDL	High-Density Lipoprotein
HOMA-IR	Homeostatic Model Assessment of Insulin Resistance
hs-CRP	High-Sensitivity C-Reactive Protein
IFC	Intrahepatic Fat Content
IL-10	Interleukin-10
IL-6	Interleukin-6
IL-8	Interleukin-8
IL-1β	Interleukin-1 Beta
IL-7A	Interleukin-7 Alpha
LDL	Low-Density Lipoprotein
MASH	Metabolic Dysfunction-Associated Steatohepatitis
MASL	Metabolic Dysfunction-Associated Steatotic Liver
MASLD	Metabolic Dysfunction-Associated Steatotic Liver Disease
MDA	Malondialdehyde
MICOL Study	Multicenter Italian Study on Cholelithiasis
MUFA	Monounsaturated Fatty Acids
NAFLD	Non-Alcoholic Fatty Liver Disease
NF-κB	Nuclear Factor kappa-light-chain-enhancer of activated B cells
NUTRIHEP Study	Nutrition and Hepatic Health Study
oxLDL	Oxidized Low-Density Lipoprotein
PUFA	Polyunsaturated Fatty Acids
SOD	Superoxide Dismutase
SREBP	Sterol Regulatory Element-Binding Protein
TBARS	Thiobarbituric Acid Reactive Substances
TC	Total Cholesterol
TG	Triglycerides
TLR4	Toll-Like Receptor 4
TNF-α	Tumor Necrosis Factor Alpha
WC	Waist Circumference
WELCOM Study	Western-Eastern Liver Cardio-Metabolic Study (confirm if referring to specific dataset)

## References

[1] Riazi K., Azhari H., Charette J.H., Underwood F.E., King J.A., Afshar E.E., Swain M.G., Congly S.E., Kaplan G.G., Shaheen A.-A. (2022). The prevalence and incidence of NAFLD worldwide: A systematic review and meta-analysis. Lancet Gastroenterol. Hepatol..

[2] Younossi Z.M., Kalligeros M., Henry L. (2024). Epidemiology of metabolic dysfunction-associated steatotic liver disease. Clin. Mol. Hepatol..

[3] Eslam M., Newsome P.N., Sarin S.K., Anstee Q.M., Targher G., Romero-Gomez M., Zelber-Sagi S., Wai-Sun Wong V., Dufour J.-F., Schattenberg J.M. (2020). A new definition for metabolic dysfunction-associated fatty liver disease: An international expert consensus statement. J. Hepatol..

[4] Tacke F., Horn P., Wong V.W.-S., Ratziu V., Bugianesi E., Francque S., Zelber-Sagi S., Valenti L., Roden M., Schick F. (2024). EASL–EASD–EASO Clinical Practice Guidelines on the management of metabolic dysfunction-associated steatotic liver disease (MASLD). J. Hepatol..

[5] Buzzetti E., Pinzani M., Tsochatzis E.A. (2016). The multiple-hit pathogenesis of non-alcoholic fatty liver disease (NAFLD). Metabolism.

[6] Kim Y., Rydqvist P., Ramezani T., Haas J.S., Bantel H., Buggisch P., Geier A., Hofmann W.-P., Mauss S., Roeb E. (2025). Metabolic Dysfunction–Associated Steatohepatitis Diagnosis and Management in Germany: Insights from an Expert Consensus Panel. Liver Int..

[7] Zeng X.-F., Varady K.A., Wang X.-D., Targher G., Byrne C.D., Tayyem R., Latella G., Bergheim I., Valenzuela R., George J. (2024). The role of dietary modification in the prevention and management of metabolic dysfunction-associated fatty liver disease: An international multidisciplinary expert consensus. Metabolism.

[8] Tosti V., Bertozzi B., Fontana L. (2018). Health Benefits of the Mediterranean Diet: Metabolic and Molecular Mechanisms. J. Gerontol. Ser. A Biol. Sci. Med. Sci..

[9] Bucciantini M., Leri M., Nardiello P., Casamenti F., Stefani M. (2021). Olive Polyphenols: Antioxidant and Anti-Inflammatory Properties. Antioxidants.

[10] Reyes-Goya C., Santana-Garrido Á., Espinosa-Martín P., Vázquez C.M., Mate A. (2024). Wild and cultivated olive trees: Nutraceutical insights of extra virgin olive oils in cardiovascular and ocular diseases. Biochim. Biophys. Acta BBA Mol. Basis Dis..

[11] Jimenez-Lopez C., Carpena M., Lourenço-Lopes C., Gallardo-Gomez M., Lorenzo J.M., Barba F.J., Prieto M.A., Simal-Gandara J. (2020). Bioactive Compounds and Quality of Extra Virgin Olive Oil. Foods.

[12] Assy N., Nassar F., Nasser G., Grosovski M. (2009). Olive oil consumption and non-alcoholic fatty liver disease. World J. Gastroenterol..

[13] Foscolou A., Critselis E., Panagiotakos D. (2018). Olive oil consumption and human health: A narrative review. Maturitas.

[14] Handu D., Moloney L., Wolfram T., Ziegler P., Acosta A., Steiber A. (2016). Academy of Nutrition and Dietetics Methodology for Conducting Systematic Reviews for the Evidence Analysis Library. J. Acad. Nutr. Diet..

[15] Myers E., Parrott J., Cummins D., Splett P. (2011). Funding Source and Research Report Quality in Nutrition Practice-Related Research. PLoS ONE.

[16] Mondon C., Tan P.Y., Chan C.L., Tran T.N., Gong Y.Y. (2024). Prevalence, determinants, intervention strategies and current gaps in addressing childhood malnutrition in Vietnam: A systematic review. BMC Public Health.

[17] Kenneally S., Sier J.H., Moore J.B. (2017). Efficacy of dietary and physical activity intervention in non-alcoholic fatty liver disease: A systematic review. BMJ Open Gastroenterol..

[18] Kanwal F., Neuschwander-Tetri B.A., Loomba R., Rinella M.E. (2024). Metabolic dysfunction–associated steatotic liver disease: Update and impact of new nomenclature on the American Association for the Study of Liver Diseases practice guidance on nonalcoholic fatty liver disease. Hepatology.

[19] Hsu C.L., Loomba R. (2024). From NAFLD to MASLD: Implications of the new nomenclature for preclinical and clinical research. Nat. Metab..

[20] Rinella M.E., Lazarus J.V., Ratziu V., Francque S.M., Sanyal A.J., Kanwal F., Romero D., Abdelmalek M.F., Anstee Q.M., Arab J.P. (2023). A multisociety Delphi consensus statement on new fatty liver disease nomenclature. J. Hepatol..

[21] Rezaei S., Akhlaghi M., Sasani M.R., Barati Boldaji R. (2019). Olive oil lessened fatty liver severity independent of cardiometabolic correction in patients with non-alcoholic fatty liver disease: A randomized clinical trial. Nutrition.

[22] Sofi F., Giangrandi I., Cesari F., Corsani I., Abbate R., Gensini G.F., Casini A. (2010). Effects of a 1-year dietary intervention with n-3 polyunsaturated fatty acid-enriched olive oil on non-alcoholic fatty liver disease patients: A preliminary study. Int. J. Food Sci. Nutr..

[23] Patti A.M., Carruba G., Cicero A.F.G., Banach M., Nikolic D., Giglio R.V., Terranova A., Soresi M., Giannitrapani L., Montalto G. (2020). Daily Use of Extra Virgin Olive Oil with High Oleocanthal Concentration Reduced Body Weight, Waist Circumference, Alanine Transaminase, Inflammatory Cytokines and Hepatic Steatosis in Subjects with the Metabolic Syndrome: A 2-Month Intervention Study. Metabolites.

[24] Pintó X., Fanlo-Maresma M., Corbella E., Corbella X., Mitjavila M.T., Moreno J.J., Casas R., Estruch R., Corella D., Bulló M. (2019). A Mediterranean Diet Rich in Extra-Virgin Olive Oil Is Associated with a Reduced Prevalence of Nonalcoholic Fatty Liver Disease in Older Individuals at High Cardiovascular Risk. J. Nutr..

[25] Nigam P., Bhatt S., Misra A., Chadha D.S., Vaidya M., Dasgupta J., Pasha Q.M.A. (2014). Effect of a 6-Month Intervention with Cooking Oils Containing a High Concentration of Monounsaturated Fatty Acids (Olive and Canola Oils) Compared with Control Oil in Male Asian Indians with Nonalcoholic Fatty Liver Disease. Diabetes Technol. Ther..

[26] Kruse M., Kemper M., Gancheva S., Osterhoff M., Dannenberger D., Markgraf D., Machann J., Hierholzer J., Roden M., Pfeiffer A.F.H. (2020). Dietary Rapeseed Oil Supplementation Reduces Hepatic Steatosis in Obese Men—A Randomized Controlled Trial. Mol. Nutr. Food Res..

[27] Tobin D., Brevik-Andersen M., Qin Y., Innes J.K., Calder P.C. (2018). Evaluation of a High Concentrate Omega-3 for Correcting the Omega-3 Fatty Acid Nutritional Deficiency in Non-Alcoholic Fatty Liver Disease (CONDIN). Nutrients.

[28] Shidfar F., Bahrololumi S.S., Doaei S., Mohammadzadeh A., Gholamalizadeh M., Mohammadimanesh A. (2018). The Effects of Extra Virgin Olive Oil on Alanine Aminotransferase, Aspartate Aminotransferase, and Ultrasonographic Indices of Hepatic Steatosis in Nonalcoholic Fatty Liver Disease Patients Undergoing Low Calorie Diet. Can. J. Gastroenterol. Hepatol..

[29] Cueto-Galán R., Barón F.J., Valdivielso P., Pintó X., Corbella E., Gómez-Gracia E., Wärnberg J. (2017). Changes in fatty liver index after consuming a Mediterranean diet: 6-Year follow-up of the PREDIMED-Malaga trial. Med. Clín. Engl. Ed..

[30] Yahay M., Heidari Z., Allameh Z., Amani R. (2021). The effects of canola and olive oils consumption compared to sunflower oil, on lipid profile and hepatic steatosis in women with polycystic ovarian syndrome: A randomized controlled trial. Lipids Health Dis..

[31] Keshk W., Ziada D., Soliman S., EL-Kalla F. (2022). The Effect of a Hypocaloric Diet Containing Olive Oil on Hepatic Steatosis Grading Using Tissue Elastography: A Randomized Controlled Trial. Afro-Egypt. J. Infect. Endem. Dis..

[32] Scorletti E., Bhatia L., McCormick K.G., Clough G.F., Nash K., Hodson L., Moyses H.E., Calder P.C., Byrne C.D., Sheron N. (2014). Effects of purified eicosapentaenoic and docosahexaenoic acids in nonalcoholic fatty liver disease: Results from the WELCOME* study. Hepatology.

[33] Quetglas-Llabrés M.M., Monserrat-Mesquida M., Bouzas C., García S., Argelich E., Casares M., Ugarriza L., Llompart I., Tur J.A., Sureda A. (2024). Impact of Adherence to the Mediterranean Diet on Antioxidant Status and Metabolic Parameters in NAFLD Patients: A 24-Month Lifestyle Intervention Study. Antioxidants.

[34] George E.S., Reddy A., Nicoll A.J., Ryan M.C., Itsiopoulos C., Abbott G., Johnson N.A., Sood S., Roberts S.K., Tierney A.C. (2022). Impact of a Mediterranean diet on hepatic and metabolic outcomes in non-alcoholic fatty liver disease: The MEDINA randomised controlled trial. Liver Int..

[35] Ristic-Medic D., Kovacic M., Takic M., Arsic A., Petrovic S., Paunovic M., Jovicic M., Vucic V. (2021). Calorie-Restricted Mediterranean and Low-Fat Diets Affect Fatty Acid Status in Individuals with Nonalcoholic Fatty Liver Disease. Nutrients.

[36] Marin-Alejandre B.A., Abete I., Cantero I., Monreal J.I., Elorz M., Herrero J.I., Benito-Boillos A., Quiroga J., Martinez-Echeverria A., Uriz-Otano J.I. (2019). The Metabolic and Hepatic Impact of Two Personalized Dietary Strategies in Subjects with Obesity and Nonalcoholic Fatty Liver Disease: The Fatty Liver in Obesity (FLiO) Randomized Controlled Trial. Nutrients.

[37] Kaliora A.C., Gioxari A., Kalafati I.P., Diolintzi A., Kokkinos A., Dedoussis G.V. (2019). The Effectiveness of Mediterranean Diet in Nonalcoholic Fatty Liver Disease Clinical Course: An Intervention Study. J. Med. Food.

[38] Barrea L., Verde L., Savastano S., Colao A., Muscogiuri G. (2023). Adherence to Mediterranean Diet: Any Association with NAFLD?. Antioxidants.

[39] Montemayor S., Mascaró C.M., Ugarriza L., Casares M., Llompart I., Abete I., Zulet M.Á., Martínez J.A., Tur J.A., Bouzas C. (2022). Adherence to Mediterranean Diet and NAFLD in Patients with Metabolic Syndrome: The FLIPAN Study. Nutrients.

[40] Properzi C., O’Sullivan T.A., Sherriff J.L., Ching H.L., Jeffrey G.P., Buckley R.F., Tibballs J., MacQuillan G.C., Garas G., Adams L.A. (2018). Ad Libitum Mediterranean and Low-Fat Diets Both Significantly Reduce Hepatic Steatosis: A Randomized Controlled Trial. Hepatology.

[41] Katsagoni C.N., Papatheodoridis G.V., Ioannidou P., Deutsch M., Alexopoulou A., Papadopoulos N., Papageorgiou M.-V., Fragopoulou E., Kontogianni M.D. (2018). Improvements in clinical characteristics of patients with non-alcoholic fatty liver disease, after an intervention based on the Mediterranean lifestyle: A randomised controlled clinical trial. Br. J. Nutr..

[42] Gelli C., Tarocchi M., Abenavoli L., Di Renzo L., Galli A., De Lorenzo A. (2017). Effect of a counseling-supported treatment with the Mediterranean diet and physical activity on the severity of the non-alcoholic fatty liver disease. World J. Gastroenterol..

[43] Abbate M., Mascaró C.M., Montemayor S., Barbería-Latasa M., Casares M., Gómez C., Angullo-Martinez E., Tejada S., Abete I., Zulet M.A. (2021). Energy Expenditure Improved Risk Factors Associated with Renal Function Loss in NAFLD and MetS Patients. Nutrients.

[44] Aller R., Izaola O., de la Fuente B., De Luis Román D.A. (2015). Mediterranean Diet Is Associated With Liver Histology In Patients With Non Alcoholic Fatty Liver Disease. Nutr. Hosp..

[45] Baratta F., Pastori D., Polimeni L., Bucci T., Ceci F., Calabrese C., Ernesti I., Pannitteri G., Violi F., Angelico F. (2017). Adherence to Mediterranean Diet and Non-Alcoholic Fatty Liver Disease: Effect on Insulin Resistance. Am. J. Gastroenterol..

[46] Schwingshackl L., Hoffmann G. (2012). Monounsaturated Fatty Acids and Risk of Cardiovascular Disease: Synopsis of the Evidence Available from Systematic Reviews and Meta-Analyses. Nutrients.

[47] Mancebo-Campos V., Salvador M.D., Fregapane G. (2023). EFSA Health Claims-Based Virgin Olive Oil Shelf-Life. Antioxidants.

[48] George E.S., Marshall S., Mayr H.L., Trakman G.L., Tatucu-Babet O.A., Lassemillante A.-C.M., Bramley A., Reddy A.J., Forsyth A., Tierney A.C. (2019). The effect of high-polyphenol extra virgin olive oil on cardiovascular risk factors: A systematic review and meta-analysis. Crit. Rev. Food Sci. Nutr..

[49] Shabalala S.C., Dludla P.V., Mabasa L., Kappo A.P., Basson A.K., Pheiffer C., Johnson R. (2020). The effect of adiponectin in the pathogenesis of non-alcoholic fatty liver disease (NAFLD) and the potential role of polyphenols in the modulation of adiponectin signaling. Biomed. Pharmacother..

[50] Bouzas C., Bibiloni M.M., Julibert A., Ruiz-Canela M., Salas-Salvadó J., Corella D., Zomeño M.D., Romaguera D., Vioque J., Alonso-Gómez Á.M. (2020). Adherence to the Mediterranean Lifestyle and Desired Body Weight Loss in a Mediterranean Adult Population with Overweight: A PREDIMED-Plus Study. Nutrients.

[51] Martínez-González M.A., García-Arellano A., Toledo E., Salas-Salvadó J., Buil-Cosiales P., Corella D., Covas M.I., Schröder H., Arós F., Gómez-Gracia E. (2012). A 14-Item Mediterranean Diet Assessment Tool and Obesity Indexes among High-Risk Subjects: The PREDIMED Trial. PLoS ONE.

[52] Panagiotakos D.B., Pitsavos C., Arvaniti F., Stefanadis C. (2007). Adherence to the Mediterranean food pattern predicts the prevalence of hypertension, hypercholesterolemia, diabetes and obesity, among healthy adults; the accuracy of the MedDietScore. Prev. Med..

[53] Schröder H., Fitó M., Estruch R., Martínez-González M.A., Corella D., Salas-Salvadó J., Lamuela-Raventós R., Ros E., Salaverría I., Fiol M. (2011). A short screener is valid for assessing Mediterranean diet adherence among older Spanish men and women. J. Nutr..

[54] Guasch-Ferré M., Willett W.C. (2021). The Mediterranean diet and health: A comprehensive overview. J. Intern. Med..

[55] Becerra-Tomás N., Blanco Mejía S., Viguiliouk E., Khan T., Kendall C.W.C., Kahleova H., Rahelić D., Sievenpiper J.L., Salas-Salvadó J. (2020). Mediterranean diet, cardiovascular disease and mortality in diabetes: A systematic review and meta-analysis of prospective cohort studies and randomized clinical trials. Crit. Rev. Food Sci. Nutr..

[56] Sebastian S.A., Padda I., Johal G. (2024). Long-term impact of mediterranean diet on cardiovascular disease prevention: A systematic review and meta-analysis of randomized controlled trials. Curr. Probl. Cardiol..

[57] Zhang Y., Yang Y., Huang Q., Zhang Q., Li M., Wu Y. (2023). The effectiveness of lifestyle interventions for diabetes remission on patients with type 2 diabetes mellitus: A systematic review and meta-analysis. Worldviews Evid. Based Nurs..

[58] Esposito K., Kastorini C.-M., Panagiotakos D.B., Giugliano D. (2013). Mediterranean diet and metabolic syndrome: An updated systematic review. Rev. Endocr. Metab. Disord..

[59] Hansrivijit P., Oli S., Khanal R., Ghahramani N., Thongprayoon C., Cheungpasitporn W. (2020). Mediterranean diet and the risk of chronic kidney disease: A systematic review and meta-analysis. Nephrol. Carlton Vic.

[60] Charkviani M., Thongprayoon C., Tangpanithandee S., Krisanapan P., Miao J., Mao M.A., Cheungpasitporn W. (2022). Effects of Mediterranean Diet, DASH Diet, and Plant-Based Diet on Outcomes among End Stage Kidney Disease Patients: A Systematic Review and Meta-Analysis. Clin. Pract..

[61] Soltani S., Jayedi A. (2022). Adherence to healthy dietary pattern and risk of kidney disease: A systematic review and meta-analysis of observational studies. Int. J. Vitam. Nutr. Res. Int. Z. Vitam.-Ernahrungsforschung J. Int. Vitaminol. Nutr..

[62] Nucci D., Sommariva A., Degoni L.M., Gallo G., Mancarella M., Natarelli F., Savoia A., Catalini A., Ferranti R., Pregliasco F.E. (2024). Association between Mediterranean diet and dementia and Alzheimer disease: A systematic review with meta-analysis. Aging Clin. Exp. Res..

[63] van den Brink A.C., Brouwer-Brolsma E.M., Berendsen A.A.M., van de Rest O. (2019). The Mediterranean, Dietary Approaches to Stop Hypertension (DASH), and Mediterranean-DASH Intervention for Neurodegenerative Delay (MIND) Diets Are Associated with Less Cognitive Decline and a Lower Risk of Alzheimer’s Disease-A Review. Adv. Nutr..

[64] Solch R.J., Aigbogun J.O., Voyiadjis A.G., Talkington G.M., Darensbourg R.M., O’Connell S., Pickett K.M., Perez S.R., Maraganore D.M. (2022). Mediterranean diet adherence, gut microbiota, and Alzheimer’s or Parkinson’s disease risk: A systematic review. J. Neurol. Sci..

[65] Morze J., Danielewicz A., Przybyłowicz K., Zeng H., Hoffmann G., Schwingshackl L. (2021). An updated systematic review and meta-analysis on adherence to mediterranean diet and risk of cancer. Eur. J. Nutr..

[66] González-Palacios Torres C., Barrios-Rodríguez R., Muñoz-Bravo C., Toledo E., Dierssen T., Jiménez-Moleón J.J. (2023). Mediterranean diet and risk of breast cancer: An umbrella review. Clin. Nutr. Edinb. Scotl..

[67] Zupo R., Castellana F., Piscitelli P., Crupi P., Desantis A., Greco E., Severino F.P., Pulimeno M., Guazzini A., Kyriakides T.C. (2023). Scientific evidence supporting the newly developed one-health labeling tool “Med-Index”: An umbrella systematic review on health benefits of mediterranean diet principles and adherence in a planeterranean perspective. J. Transl. Med..

[68] Rajewski P., Cieściński J., Rajewski P., Suwała S., Rajewska A., Potasz M. (2025). Dietary Interventions and Physical Activity as Crucial Factors in the Prevention and Treatment of Metabolic Dysfunction-Associated Steatotic Liver Disease. Biomedicines.

[69] WHO Updates Guidelines on Fats and Carbohydrates. https://www.who.int/news/item/17-07-2023-who-updates-guidelines-on-fats-and-carbohydrates.

[70] Green C.J., Hodson L. (2014). The Influence of Dietary Fat on Liver Fat Accumulation. Nutrients.

[71] Tedesco C.C., Bonfiglio C., Notarnicola M., Rendina M., Castellaneta A., Di Leo A., Giannelli G., Fontana L. (2023). High Extra Virgin Olive Oil Consumption Is Linked to a Lower Prevalence of NAFLD with a Prominent Effect in Obese Subjects: Results from the MICOL Study. Nutrients.

[72] Bonfiglio C., Cuccaro F., Campanella A., Rosso N., Tatoli R., Giannelli G., Donghia R. (2023). Effect of Intake of Extra Virgin Olive Oil on Mortality in a South Italian Cohort with and without NAFLD. Nutrients.

[73] Donghia R., Tatoli R., Campanella A., Losurdo G., Di Leo A., De Pergola G., Bonfiglio C., Giannelli G. (2024). Extra Virgin Olive Oil Reduces the Risk of Non-Alcoholic Fatty Liver Disease in Females but Not in Males: Results from the NUTRIHEP Cohort. Nutrients.

[74] Flynn M., Tierney A., Itsiopoulos C. (2023). Is Extra Virgin Olive Oil the Critical Ingredient Driving the Health Benefits of a Mediterranean Diet? A Narrative Review. Nutrients.

[75] Seidita A., Soresi M., Giannitrapani L., Di Stefano V., Citarrella R., Mirarchi L., Cusimano A., Augello G., Carroccio A., Iovanna J.L. (2022). The clinical impact of an extra virgin olive oil enriched mediterranean diet on metabolic syndrome: Lights and shadows of a nutraceutical approach. Front. Nutr..

[76] Lucas L., Russell A., Keast R. (2011). Molecular mechanisms of inflammation. Anti-inflammatory benefits of virgin olive oil and the phenolic compound oleocanthal. Curr. Pharm. Des..

[77] Sato K., Arai H., Mizuno A., Fukaya M., Sato T., Koganei M., Sasaki H., Yamamoto H., Taketani Y., Doi T. (2007). Dietary palatinose and oleic acid ameliorate disorders of glucose and lipid metabolism in Zucker fatty rats. J. Nutr..

[78] Paniagua J.A., Gallego de la Sacristana A., Romero I., Vidal-Puig A., Latre J.M., Sanchez E., Perez-Martinez P., Lopez-Miranda J., Perez-Jimenez F. (2007). Monounsaturated fat-rich diet prevents central body fat distribution and decreases postprandial adiponectin expression induced by a carbohydrate-rich diet in insulin-resistant subjects. Diabetes Care.

[79] Njei B., Ameyaw P., Al-Ajlouni Y., Njei L.-P., Boateng S. (2024). Diagnosis and Management of Lean Metabolic Dysfunction-Associated Steatotic Liver Disease (MASLD): A Systematic Review. Cureus.

[80] Sato-Espinoza K., Chotiprasidhi P., Huaman M.R., Díaz-Ferrer J. (2024). Update in lean metabolic dysfunction-associated steatotic liver disease. World J. Hepatol..

